# Doping of vanadium to nanocrystalline diamond films by hot filament chemical vapor deposition

**DOI:** 10.1186/1556-276X-7-441

**Published:** 2012-08-08

**Authors:** Yaozhong Zhang, Liying Zhang, Jiang Zhao, Liang Wang, Gang Zhao, Yafei Zhang

**Affiliations:** 1 Key Laboratory for Thin Film and Microfabrication of the Ministry of Education, Institute of Micro/Nano Science and Technology, Shanghai Jiao Tong University, Shanghai, 200030, People's Republic of China

**Keywords:** Nanocrystalline diamond, Vanadium dopant, Donor state levels, Structural distortion toleration

## Abstract

Doping an impure element with a larger atomic volume into crystalline structure of buck crystals is normally blocked because the rigid crystalline structure could not tolerate a larger distortion. However, this difficulty may be weakened for nanocrystalline structures. Diamonds, as well as many semiconductors, have a difficulty in effective doping. Theoretical calculations carried out by DFT indicate that vanadium (V) is a dopant element for the n-type diamond semiconductor, and their several donor state levels are distributed between the conduction band and middle bandgap position in the V-doped band structure of diamond. Experimental investigation of doping vanadium into nanocrystalline diamond films (NDFs) was first attempted by hot filament chemical vapor deposition technique. Acetone/H_2_ gas mixtures and vanadium oxytripropoxide (VO(OCH_2_CH_2_CH_3_)_3_) solutions of acetone with V and C elemental ratios of 1:5,000, 1:2,000, and 1:1,000 were used as carbon and vanadium sources, respectively. The resistivity of the V-doped NDFs decreased two orders with the increasing V/C ratios.

## Background

Doping an impure element with a larger atomic volume into crystalline structure of buck crystals is normally blocked because the rigid structure could not tolerate a larger distortion. However, this difficulty may be overcome to some extent for nanocrystalline materials due to its weakened structure with large a surface-to-volume ratio. As a typical example, carbon nanotubes have been doped with silicon, sulfur [1], etc. Diamond is a super-functional material with many promising properties, which has been utilized in many commercial applications such as electrochemical electrodes, heterojunction, photodiode, radiation detectors, and high-frequency SAW devices [2-5]. Diamond films have high electrical resistivity when undoped and could be effectively p-type doped by boron [6,7]. However, the realization of n-type doping of diamond films, based on device application, has met a serious obstacle of tough impurity doping problem [8]. The ideal n-type diamond films for electronic applications are hard to be acquired in experiments with the doping of Li, Na, N, P, S, As, Sb, etc [9-12]. Finding a well-established substitutional donor for n-type diamond films is a worldwide issue, owing to the extremely small lattice space between C-C atoms within the diamond structure. Previous studies on most impurity elements which have been reported are in the main group, and the subgroup element is rarely seen in papers because of its complicated atom structure with larger atomic sizes and its acquisition difficulty.

The research purpose of this paper is to perform first principle calculations to study the electronic properties of V-doped diamond, and attempt to dope vanadium into nanocrystalline diamond films (NDFs), and to test if the V element can be used as dopant species which could be carried through bubbling in acetone by hydrogen during chemical vapor deposition process.

## Methods

**Figure 1:**
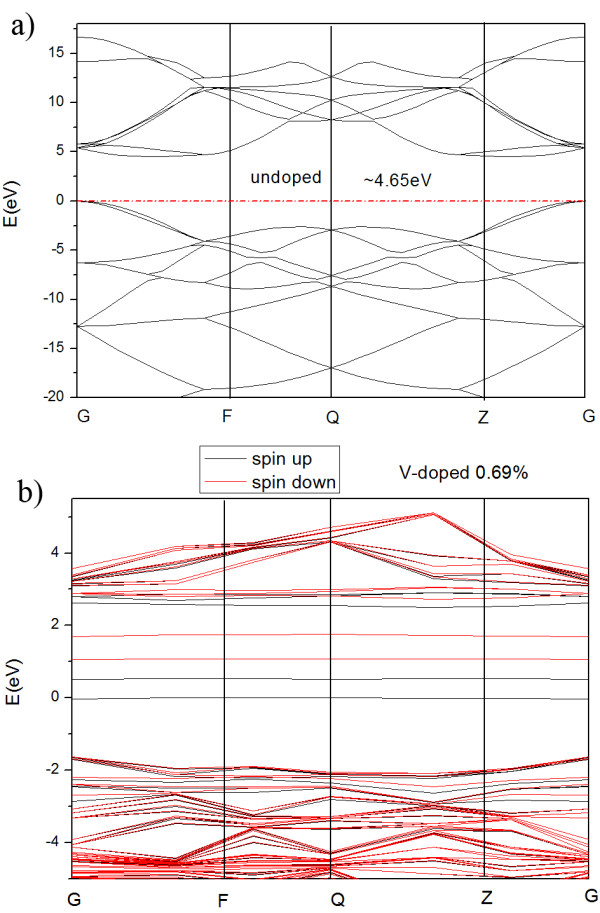
The band structures of undoped and V-doped diamonds.

First principle calculations were performed to study the electronic properties of V-doped diamond with a doping concentration of 0.69%. The calculations are carried out by DFT implemented in the Dmol3 package [13,14]. One V atom is used to substitute a carbon in the diamond supercell with 144 atoms. The structure considered is fully relaxed to an accuracy where the self-consistent field procedure was done with a convergence criterion of 10^−5^ a.u. The all-electron Kohn-Sham wave functions were expanded in the local atomic orbital (double numerical polarization) basis set and generalized gradient approximation of Perdew-Burke-Ernzerhof for the exchange-correlation potential [15]. The Monkhorst-Pack scheme is used in the Brillouin zone with 3 × 3 × 3 for all the geometry optimization and total energies' calculations [16]. After structural relaxation, the three adjacent V-C bonds were extended from about 1.544 to 1.804 Å, respectively, owing to the large atomic radius of vanadium. In addition, three bond angles of C-V-C changed from 109.5° to 110.2°, respectively. The detailed changes of the atomic structure are collected in Table 1. They increase approximately 16.8% for bond length and 0.6% for bond angles as compared with those of the unrelaxed structure. The structure of V-doped diamond has a strong distortion which may suggest the difficulty of doping V atom into the diamond. The band structure of V-doped diamond is shown in Figure 1. It is clear that there are several n-type local states distributed between the conduction band and the middle bandgap position in the V-doped band structure of diamond. Such local states (impurity level) originate from the contribution of spin-up and spin-down electrons which make the band structure complex. Furthermore, the presence of the impurity level may make a complex electron transition from valence band to conduction band under an external electronic field which is favorable for the amelioration of the electrical conductivity of diamond. From the results of theoretical calculations, it is encouraged to ameliorate the electrical conductivity of diamond by V doping.

**Table 1:** Changes of the atomic structure of V-doped diamond

**Type of diamond film**	**Bond length (Å)**	**Bond angle (degrees)**
Undoped	1.544, 1.544, and 1.544	109.5, 109.5, and 109.5
V-doped	1.804, 1.804, and 1.804	110.2, 110.2, and 110.2

**Figure 2:**
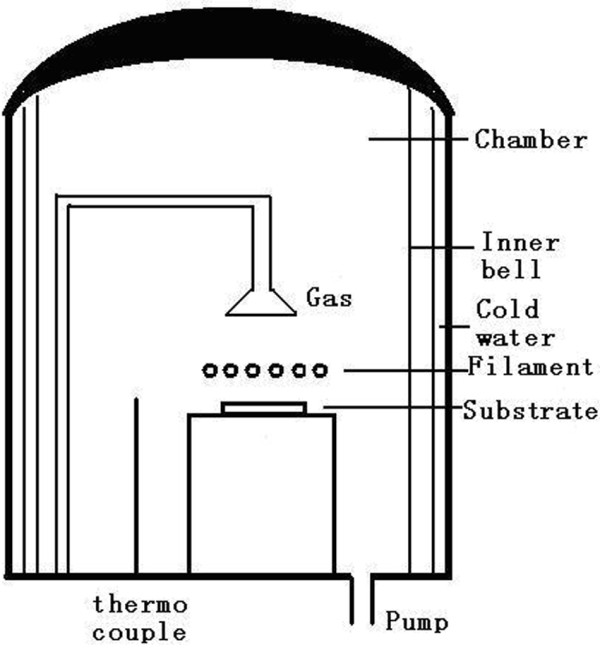
Schematic of a hot filament chemical vapor deposition reactor.

Experimental test of doping vanadium into NDFs was investigated by hot filament chemical vapor deposition (HFCVD) technique. The HFCVD NDFs were deposited on (100)-oriented silicon substrate by a conventional HFCVD system that used tantalum wire as a filament. Acetone and hydrogen (C/H = 1.5%) mixture gases (total flow 250 sccm) were used as carbon source gases and were maintained at a process pressure of 4 kPa in the chamber which was vacuumized prior to the experiment. Silicon substrates were scratched manually with diamond powder (average grain size of approximately 0.5 to 1.0 μm) for 20 min in order to enhance the nucleation density, then cleaned ultrasonically in acetone and deionized water, and finally dried in an oven at 80°C. The substrate temperature was stabilized at 850°C by maintaining the distance between the Ta filament and the substrate with 7 mm. The deposition time was 3 h, with a diamond deposition rate of about 1 μm/h. Figure 2 shows the schematic of the HFCVD reactor.

For doping experiments, vanadyl acetylacetonate (VO(C_5_H_7_O_2_)_2_) powder, which was dissolved in acetone, was used at first as the source of vanadium for dissolubility and less toxicity. However, we found that during the experiment, the color of the liquid mixture changed from light green to dark green, and we measured the weight of the residual vanadyl acetylacetonate only to find that it was almost the same as that of the powder prior to deposition. It clearly means that little powder was carried out into the chamber. Based on the above consideration, the vanadium source selected was vanadium oxytripropoxide (VO(OCH_2_CH_2_CH_3_)_3_), which was in a liquid state at room temperature and was diffluent in acetone. However, we also found that when the V/C ratio was 1:1,000, it had already been saturated, so this ratio was the maximum in the experiment. At this time, the color of the liquid mixture was clarified as red-brown, and it never changed throughout the process, which could confirm that V was successfully being introduced into the chamber in sufficient amounts.

Different dilutions were used to vary the V/C ratio in the reactor gas phase, which was 0 (sample 1, undoped), 1:5,000 (sample 2), 1:2,000 (sample 3), 1:1,000 (sample 4). All these samples were grown on 2 × 2-cm^2^ silicon substrates, and the depositional conditions were also the same.

The deposited NDF morphology had been characterized using field emission scanning electron microscopy (ULTRA 55, Carl Zeiss AG, Oberkochen, Germany). Laser Raman spectroscopy (325-nm excitation) was used to evaluate the average sizes of nanocrystals in NDFs. Resistivity measurements were carried out by an Agilent voltmeter (Agilent Technologies, Inc., Santa Clara, CA, USA). X-ray fluorescence measurements were to probe the V concentration in the sample films.

## Results and discussion

**Figure 3:**
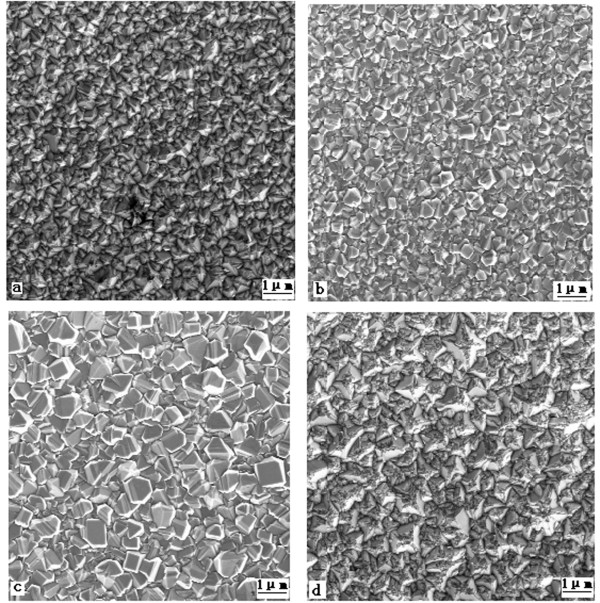
**SEM images of NDF samples for different V/C ratios.** (**a**) Undoped, (**b**) 1:5,000, (**c**) 1:2,000, and (**d**) 1:1,000.

Scanning electron microscopy (SEM) micrographs of the films deposited under the mentioned conditions are shown in Figure 3a,b,c,d, which corresponds to samples 1, 2, 3, and 4, respectively. A nanocrystalline morphology is intuitively observed. Morphology difference at the NDF sample surface with different vanadium doping concentrations cannot be obviously distinguished.

**Figure 4:**
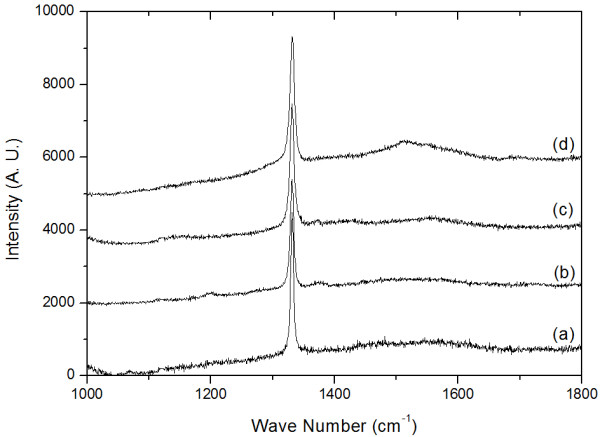
**Raman spectra of NDF samples for different V/C ratios.** (**a**) Undoped, (**b**) 1:5,000, (**c**) 1:2,000, and (**d**) 1:1,000.

Raman scattering spectra features pertaining to different carbon phases with the effects of different V/C ratios are observed in Figure 4. The characteristic Raman peak of nanocrystalline diamond films appears around 1,331.61 cm^−1^ with full width at half maximum (FWHM), which is scattered from the diamond phase. For samples 1, 2, 3, and 4, the FWHM are 6.2, 8.1, 9.2, and 11.7 cm^−1^, respectively. By estimation of crystal size from that Raman scattering spectra [17], the grain sizes of the nanocrystalline diamond films are 11.2, 8.6, 7.6, and 6.0 nm. This result indicated that the nanocrystalline structures are not changed significantly due to the increasing V/C ratio from 0 to 1:1,000 in the reactor gas phase.

**Figure 5:**
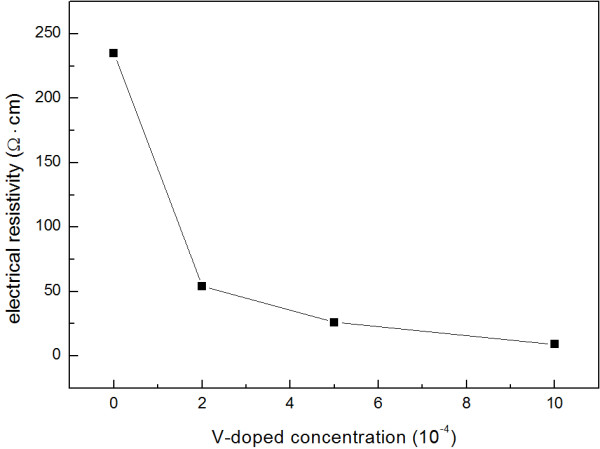
**Resistivity of NDF samples for different V/C ratios.** (**a**) Undoped, (**b**) 1:5,000, (**c**) 1:2,000, and (**d**) 1:1,000.

The resistivity values of the NDF samples correspond to the different V/C ratios in the gas phase of HFCVD which were measured at room temperature, as seen in Figure 5. All measurements were carried out between two contacts of parallel Ag electrodes with the same dimensions (0.5 mm). It can be seen that about a two-order decrease of magnitude of resistivity was induced due to the increasing V/C ratio from 0 to 1:1,000. This value is large enough for judging that it is caused by the effect of doping V. Further analysis by X-ray fluorescence spectra showed that the doping concentration of V are 0, 17, 56, 98 ppm which correspond to samples 1, 2, 3, and 4, respectively. These measurement results have supposed the realistic doping of V into the nanocrystalline diamond structure, but the doping concentration is suppressed to a limited extent.

## Conclusions

Theoretical calculation reveals that V is a dopant element for the n-type diamond semiconductor, but strong structural distortion is a significant difficulty for doping V into the diamond lattice. Experiments have demonstrated a way to doping V into NDFs in HFCVD conditions. The results from experiments indicated that doping an impure element with a larger atomic volume into the nanocrystalline-structured materials may be a possible way to synthesize normally difficult-to-dope semiconductors and that nanocrystalline structures could tolerate impurity induced by larger distortions.

## Competing interests

The authors declare that they have no competing interests.

## Authors' contributions

YZZ performed the theoretic calculations and experiments, and drafted the manuscript. LZ guided the idea and the experiments, and checked the figures. JZ performed the experiments, checked the figures, and finalized the manuscript. LW guided the theoretic calculations. GZ performed the experiments. YFZ proposed the idea and guided the scheme. All authors read and approved the final manuscript.
